# Nickel Allergy in Cardiothoracic Surgery: A Case Report

**DOI:** 10.1055/a-2862-1054

**Published:** 2026-05-19

**Authors:** Jason Sanchez, Andrew Brazier, Adeyemi Olayiwola, Uday Dandekar

**Affiliations:** 61138 University Hospitals Coventry and Warwickshire NHS Trust, Coventry, England, United Kingdom

**Keywords:** cardiovascular surgery, aortic valve, TAVI, CABG, cardiac catheterization, aortic disease

## Abstract

**Background:**

Metal hypersensitivity represents a rare but important challenge in cardiothoracic surgery, particularly during prosthesis selection for aortic valve replacement.

**Case Description:**

A 63-year-old woman with critical bicuspid aortic stenosis and moderate left ventricular dysfunction was found to have severe hypersensitivity to multiple metal alloys, including nickel, palladium, and iridium. Following multidisciplinary evaluation, standard surgical aortic valve replacement was considered unsafe. A transfemoral transcatheter aortic valve implantation using a cobalt–chromium transcatheter valve was successfully performed without complications.

**Conclusion:**

This case highlights the importance of individualized, multidisciplinary decision-making when managing patients with significant metal hypersensitivity.

## Introduction

Aortic stenosis (AS) is a common valvular heart disease associated with significant morbidity and mortality if untreated. Surgical aortic valve replacement (SAVR) remains the gold-standard treatment for severe AS in younger patients less than 70 years. However, transcatheter aortic valve implantation (TAVI) remains a viable option for elderly and selected high-risk patients. Metal hypersensitivity presents unique challenges in prosthetic valve selection, particularly when patients have confirmed allergies to nickel and palladium—metal alloys commonly present in sternal wires (8–12% nickel), pacing wires, and many prosthetic valve frames. Hypersensitivity reactions may manifest as dermatitis, systemic inflammation, or, rarely, structural valve dysfunction. 1 2 3 This report presents a case with severe metal alloy allergy necessitating deviation from standard surgical treatment.

## Case Presentation

A 63-year-old woman presented with acute shortness of breath, dry cough, orthopnea, and respiratory distress following recent treatment for a chest infection. She also reported exertional chest pain. Her past medical history included asthma, type 2 diabetes mellitus, osteoarthritis, rheumatoid arthritis, chronic pain, diverticulosis, and a significant smoking history. On admission, she required noninvasive ventilation for type 2 respiratory failure. Clinical examination demonstrated an ejection systolic murmur with an absent second heart sound and an elevated jugular venous pressure measuring approximately 13 cm H _2_ O (approximately 8 cm above the sternal notch). Investigations revealed a troponin I level of 638 ng/L, lower lobe consolidation, and pulmonary edema.

**Fig. 1:**
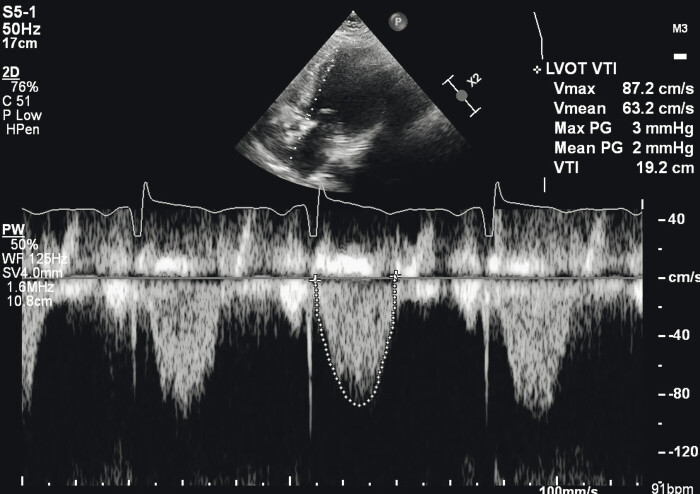
Transthoracic echocardiography demonstrating severe calcification of a bicuspid aortic valve with critical aortic stenosis, moderate left ventricular systolic dysfunction, regional wall motion abnormalities, and concentric left ventricular hypertrophy.

Transthoracic echocardiography demonstrated severe calcification of a bicuspid aortic valve with critical AS (aortic valve area: 0.65 cm ^2^ , peak velocity: 4.9 m/s), moderate left ventricular systolic dysfunction (LVEF: 40–45%), regional wall motion abnormalities, and concentric left ventricular hypertrophy ( Fig. 1 ).

Given the presentation with pulmonary edema and chest pain, urgent invasive coronary angiography was performed. This demonstrated normal left main, left anterior descending, and right coronary arteries, with a long 60% stenosis in the ostial segment of a large obtuse marginal branch of the left circumflex artery.

**Fig. 2:**
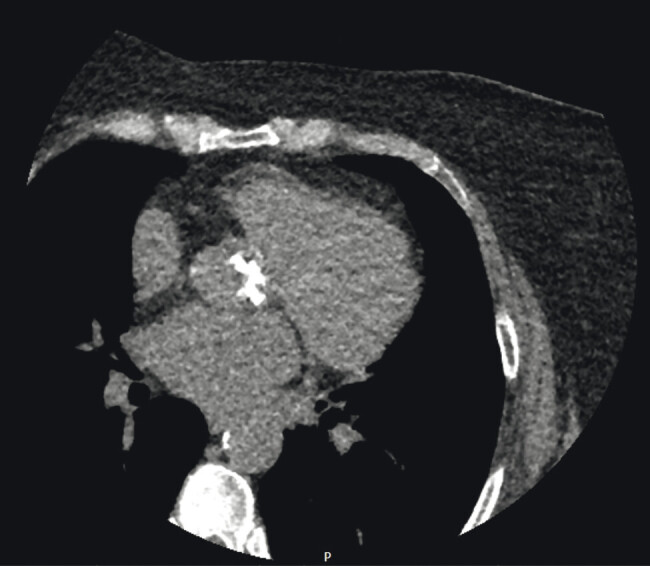
Cardiac CT coronary angiography at the level of the aortic valve demonstrating marked calcification of the bicuspid aortic valve.

A comprehensive cardiac CT assessment was subsequently performed. CT angiography confirmed a bicuspid aortic valve (Sievers type I, LCC/RCC fusion) with severe calcification of the raphe and leaflets ( Fig. 2 ).

**Fig. 3:**
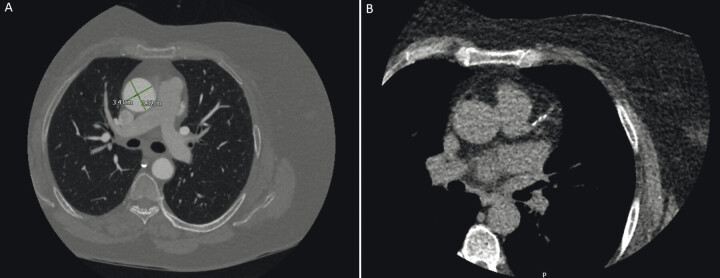
Computed tomography imaging used for procedural planning. ( **A** ) CT angiography of the thoracic aorta at the level of the pulmonary artery bifurcation demonstrates normal dimensions of the ascending aorta. ( **B** ) CT coronary angiography demonstrating mild coronary artery calcification without significant obstructive disease.

Annular dimensions measured 22.9 mm, with a sinus of Valsalva diameter of 32.4 mm (cusp-to-cusp) and an ascending aortic diameter of 33.8 mm ( Fig. 3A ).

CT coronary angiography demonstrated mild coronary calcification without significant obstructive disease ( Fig. 3B ).

Because the patient reported a history of hypersensitivity reactions to metal jewelry, a dermatology consultation was obtained, and formal patch testing was performed. Formal dermatology patch testing demonstrated strong hypersensitivity to nickel, palladium, sodium chloropalladate, and iridium—metal alloys commonly present in sternal wires, pacing wires, and prosthetic valve components.

A further multidisciplinary discussion concluded that standard SAVR posed an unacceptably high risk of hypersensitivity reaction. A transcatheter approach using a cobalt–chromium–framed Edwards Sapien 3 transcatheter valve was therefore agreed upon. In view of the patient's documented metal allergy, percutaneous coronary intervention and stent implantation were deliberately avoided.

**Fig. 4:**
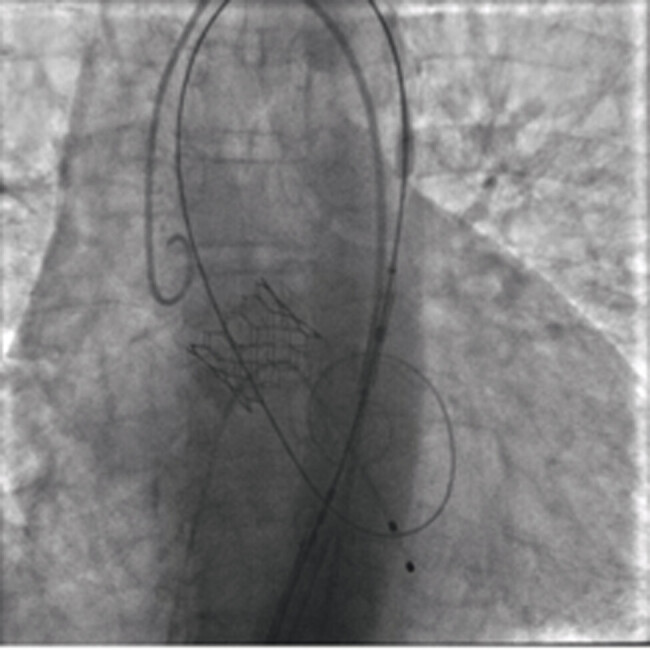
Fluoroscopic image during transfemoral transcatheter aortic valve implantation showing deployment of a 23-mm Edwards Sapien 3 valve.

The patient subsequently underwent elective transfemoral TAVI with implantation of a 23-mm Edwards Sapien 3 valve ( Fig. 4 ).

The procedure was completed successfully without complications. She made an uneventful recovery and was discharged on lifelong aspirin therapy. Follow-up in the dedicated TAVI clinic was arranged at 3 to 6 months.

## Discussion

Allergic contact dermatitis (ACD) is a delayed hypersensitivity reaction following repeated exposure to an irritant or allergen, with nickel being the most common culprit. It is the most prevalent occupational skin disease. 1 Nickel allergy, a subset of ACD, is commonly triggered by contact with jewelry, kitchen utensils, and silverware. In sensitized individuals, dietary ingestion of nickel-containing foods, such as chocolate, nuts, oats, green beans, and peas, can lead to systemic nickel allergy syndrome or chronic dermatitis. Additionally, implantable medical devices pose an increased risk of systemic hypersensitivity reactions. 1 2 4 A cross-sectional study across five European countries found that 27.0% of individuals aged 18 to 74 years exhibited at least one positive reaction to allergens from the European baseline series, with a significantly higher prevalence in women. Nickel allergy was the most common, affecting 14.5% of the population (95% CI: 13.2–15.8), followed by thiomersal (5.0%), cobalt (2.2%), and other allergens. 2 The pathophysiology of nickel allergy follows a classic T cell-mediated, delayed-type hypersensitivity response. Sensitization begins with hapten binding to a skin protein carrier, forming a hapten–protein complex that activates T cells. Subsequent antigen exposure triggers cytokine release, macrophage activation, and a localized immune response. 3 In cardiac surgery, prosthetic heart valves often contain nickel in their stent material, presenting a significant risk for allergic patients. Hypersensitivity reactions to nickel in prosthetic valves can lead to periprosthetic leaks, while stainless steel sutures used in sternotomy closure may cause sternal pruritus or pain. 4 The 2024 NACSA report, covering NHS cardiac surgery centers between April 2020 and March 2023, identified AVR as the second most common cardiac surgery, with 3,623 isolated AVRs performed in England, Wales, and Northern Ireland during 2022/23. 5 Only four cases of Nickel-related complications in cardiothoracic surgery have been documented for decades. In 1978, Lyell et al reported a patient with nickel allergy who experienced life-threatening peri-prosthetic incompetence with two successive nickel-containing mitral valve prostheses. Both valves failed to integrate properly, and a subsequent nickel-free prosthesis proved successful 22 months postimplantation, highlighting the importance of selecting nickel-free options for sensitized patients. 4 Similarly, Gordon et al described a 58-year-old woman who developed an eczematous rash on her chest after receiving nickel-containing sternotomy wires. Symptoms resolved following wire removal, with biopsy findings confirming a sarcoidal reaction. 6 Lusini et al further demonstrated the successful use of a nickel-free ON-X prosthesis in a 51-year-old woman with severe nickel allergy, utilizing Fibertape suture for sternal closure to mitigate hypersensitivity risks. 7 Dominguez-Massa et al presented a case of a 56-year-old man who developed persistent urticaria and anaphylactic shock postmitral valve repair, with prick testing confirming nickel sensitivity. The rash resolved following removal of the mitral annular ring, emphasizing the potential severity of nickel-related allergic reactions in cardiac surgery. 8 In our case, a 63-year-old woman with severe bicuspid AS (AVA: 0.65 cm ^2^ , peak velocity: 4.9 m/s), moderate LVEF (42%), and concentric hypertrophy was initially considered for AVR and coronary artery bypass grafting (CABG). However, patch testing revealed significant allergies to nickel, palladium, and sodium chloropalladate, rendering her unsuitable for conventional open-heart surgery. After extensive MDT deliberation and risk assessment, the decision was made to proceed with TAVI using a cobalt–chromium–framed Edwards Sapien 3 transcatheter valve. The procedure was successful, with no residual aortic regurgitation or postoperative complications. The patient remained clinically stable and was subsequently discharged with scheduled follow-up in the TAVI nurse clinic.

## Conclusion

This case highlights the importance of multidisciplinary collaboration and individualized decision-making when managing patients with severe AS who present with material hypersensitivities. Although SAVR remains the first-line treatment for severe AS in patients of this age and risk profile, the disclosure of a long-standing severe nickel allergy raised significant concerns regarding the safety of standard surgical approaches. Routine preoperative metal allergy screening is not recommended; however, dermatological evaluation and patch testing may be considered in patients with a history suggestive of metal hypersensitivity prior to implantation of cardiac prosthetic devices. In selected young patients with significant metal alloy hypersensitivity, TAVI may be considered an important addition to the therapeutic arsenal when standard surgical approaches carry unacceptable risk.
